# Clinical Correlates and Prognostic Value of Plasma Galectin-3 Levels in Degenerative Aortic Stenosis: A Single-Center Prospective Study of Patients Referred for Invasive Treatment

**DOI:** 10.3390/ijms18050947

**Published:** 2017-04-29

**Authors:** Beata Bobrowska, Ewa Wieczorek-Surdacka, Olga Kruszelnicka, Bernadeta Chyrchel, Andrzej Surdacki, Dariusz Dudek

**Affiliations:** 1 Second Department of Cardiology, Faculty of Medicine, Jagiellonian University Medical College, 17 Kopernika Street, 31-501 Cracow, Poland; bobrowska.beata@gmail.com (B.B.); chyrchelb@gmail.com (B.C.); mcdudek@cyfromet.pl (D.D.); 2 Second Department of Cardiology and Cardiovascular Interventions, University Hospital, 17 Kopernika Street, 31-501 Cracow, Poland; 3 Department of Nephrology, University Hospital, 15C Kopernika Street, 31-501 Cracow, Poland; esurdacka@gmail.com; 4 Department of Coronary Artery Disease and Heart Failure, John Paul II Hospital, 80 Prądnicka Street, 31-202 Cracow, Poland; olga.kruszelnicka@onet.pl

**Keywords:** galectin-3, degenerative aortic stenosis, prognostic value, mortality, invasive treatment, balloon aortic valvuloplasty

## Abstract

Galectin-3 (Gal-3), a β-galactoside-binding lectin, has been implicated in myocardial fibrosis, development of left ventricular (LV) dysfunction and transition from compensated LV hypertrophy to overt heart failure (HF), being a novel prognostic marker in HF. Risk stratification is crucial for the choice of the optimal therapy in degenerative aortic stenosis (AS), affecting elderly subjects with coexistent diseases. Our aim was to assess correlates and prognostic value of circulating Gal-3 in real-world patients with degenerative AS referred for invasive treatment. Gal-3 levels were measured at admission in 80 consecutive patients with symptomatic degenerative AS (mean age: 79 ± 8 years; aortic valve area (AVA) index: 0.4 ± 0.1 cm^2^/m^2^). The therapeutic strategy was chosen following a dedicated multidisciplinary team-oriented approach, including surgical valve replacement (*n* = 11), transcatheter valve implantation (*n* = 19), balloon aortic valvuloplasty (BAV) (*n* = 25) and optimal medical therapy (*n* = 25). Besides routine echocardiographic indices, valvulo-arterial impedance (Zva), an index of global LV afterload, was computed. There were 22 deaths over a median follow-up of 523 days. Baseline Gal-3 correlated negatively with estimated glomerular filtration rate (eGFR) (*r* = −0.61, *p* < 0.001) and was unrelated to age, symptomatic status, AVA index, LV ejection fraction, LV mass index or Zva. For the study group as a whole, Gal-3 tended to predict mortality (Gal-3 >17.8 vs. Gal-3 <17.8 ng/mL; hazard ratio (HR): 2.03 (95% confidence interval, 0.88–4.69), *p* = 0.09), which was abolished upon adjustment for eGFR (HR: 1.70 (0.61–4.73), *p* = 0.3). However, in post-BAV patients multivariate-adjusted pre-procedural Gal-3 was associated with worse survival (HR: 7.41 (1.52–36.1), *p* = 0.01) regardless of eGFR. In conclusion, the inverse eGFR–Gal-3 relationship underlies a weak association between Gal-3 and adverse outcome in patients with degenerative AS referred for invasive therapy irrespective of type of treatment employed. In contrast, pre-procedural Gal-3 appears an independent mortality predictor in high-risk AS patients undergoing BAV.

## 1. Introduction

Galectin-3 (Gal-3), a β-galactoside-binding lectin, has been implicated in myocardial fibrosis, development of left ventricular (LV) dysfunction and transition from compensated left ventricular (LV) hypertrophy to overt heart failure (HF) in various experimental HF models [1,2,3,4]. Gal-3—produced mainly by activated macrophages, but also mast cells and pericytes—induces transformation of fibroblasts into myofibroblasts, fibroblast/myofibroblast proliferation, and collagen deposition [1,5]. In HF patients, Gal-3 levels predict progression of LV remodeling [6], hospitalization for worsening HF and death irrespective of LV ejection fraction (EF) [6,7,8,9,10,11,12]. Additionally, Gal-3 concentrations are associated with incident HF and mortality in the general population [13,14,15,16]. According to recent HF guidelines [17], Gal-3 assay may be considered for improved risk stratification in both acute and chronic HF, offering additional prognostic value beyond natriuretic peptides. In contrast to natriuretic peptides, Gal-3 is considered a “slow” marker, whose levels do not change rapidly in response to compensation state [18,19].

In an early clinical report, Sharma et al. [1] described increased myocardial Gal-3 expression in patients with severe aortic stenosis (AS) and depressed EF in comparison to AS subjects with compensated LV hypertrophy. However, later clinical studies on Gal-3 in AS have been sparse. In the largest series of AS patients described to date, Gal-3 levels were independent of stenosis severity and LV mass and only weakly correlated inversely with EF [20]. Additionally, Gal-3 was unrelated to symptomatic status and did not provide prognostic information on the risk of AS-related events, mainly new-onset HF, in asymptomatic subjects [20]. Moreover, conflicting results were reported with regard to the prognostic ability of pre-procedural Gal-3 in AS patients undergoing valve replacement. In particular, pre-procedural Gal-3 either independently predicted all-cause mortality [21], was predictive only on univariate analysis [22], or lacked any relationship with survival [23]. Finally, a comparison of the prognostic effect of Gal-3 between different treatment modalities for severe AS stenosis has not been reported so far.

Risk stratification is crucial for the choice of the optimal therapy in degenerative AS, affecting mainly elderly subjects with coexistent diseases, thereby posing a therapeutic challenge for cardiac surgeons and invasive cardiologists [24]. Thus, our aim was to assess determinants and prognostic value of circulating Gal-3 in consecutive real-world patients with symptomatic degenerative AS referred for invasive treatment, including various therapeutic strategies.

## 2. Results

### 2.1. Patients’ Characteristics

Demographic, clinical and echocardiographic characteristics of AS subjects are shown in Table 1 and Table 2.

### 2.2. Correlates of Plasma Gal-3 Levels

Median Gal-3 level was 15.6 ng/mL (range, 7.5–45.6 ng/mL; interquartile range, 11.7–20.2 ng/mL) and was skewed to the right. Out of 80 subjects enrolled, 30 patients (38%)—in accordance with a previous report based on the same method of Gal-3 assay in 101 AS patients undergoing transcatheter aortic valve implantation (TAVI) [21]—had a Gal-3 level higher than the cut-off value of 17.8 ng/mL, previously proposed for risk stratification in established HF [5,11].

**Figure 1:**
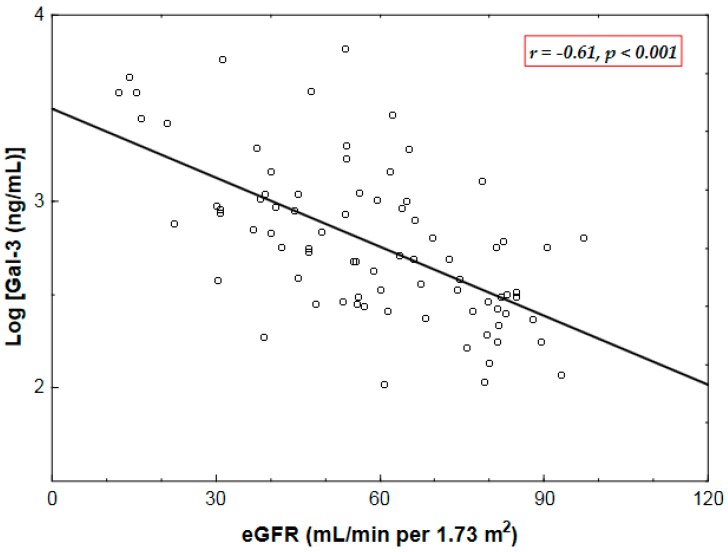
A negative correlation between natural logarithmically (log)-transformed plasma levels of galectin-3 (Gal-3) and estimated glomerular filtration rate (eGFR) in patients with aortic stenosis. *r*: Pearson’s correlation coefficient.

Baseline Gal-3 correlated negatively with estimated glomerular filtration rate (eGFR) (*r* = −0.61, *p* < 0.001) (Table 3, Figure 1) and positively with N-terminal pro-B-type natriuretic peptide (NT-proBNP) (*r* = 0.39, *p* < 0.001), being unrelated to age, symptomatic status, AVA index, transvalvular aortic pressure gradients, EF, LV mass index or valvulo-arterial impedance (Zva), a measure of global LV afterload (Table 3).

### 2.3. Prognostic Value of Plasma Gal-3 Levels

TAVI, balloon aortic valvuloplasty (BAV) and surgical valve replacement (SVR) were performed in 19, 25 and 11 patients, respectively, while 25 subjects received optimal medical therapy. Subjects who underwent BAV were significantly older compared to the remainder (Table 4).

**Figure 2:**
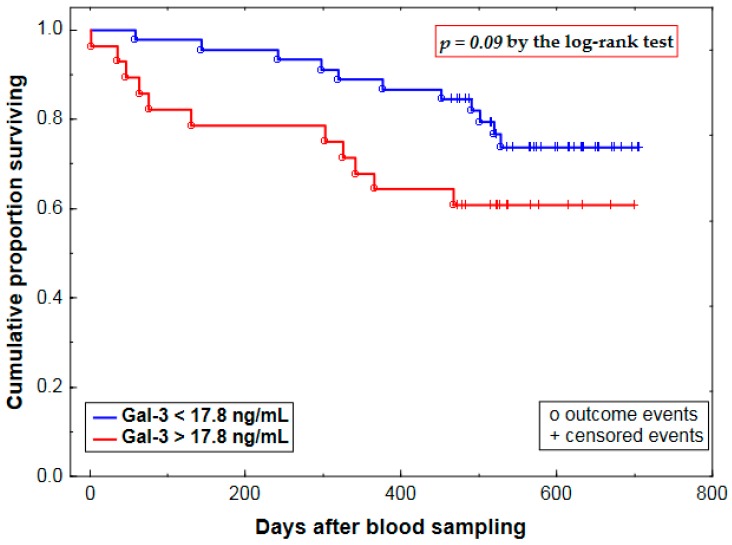
Kaplan–Meier survival curves by baseline plasma galectin-3 (Gal-3) for all patients with aortic stenosis irrespective of type of treatment employed.

In the study group as a whole, Gal-3 exhibited a borderline or weak association with all-cause mortality (22 deaths) over a median follow-up period of 523 days (Table 5, Figure 2). However, the predictive ability of Gal-3 was lost upon adjustment for eGFR (Table 5).

**Figure 3:**
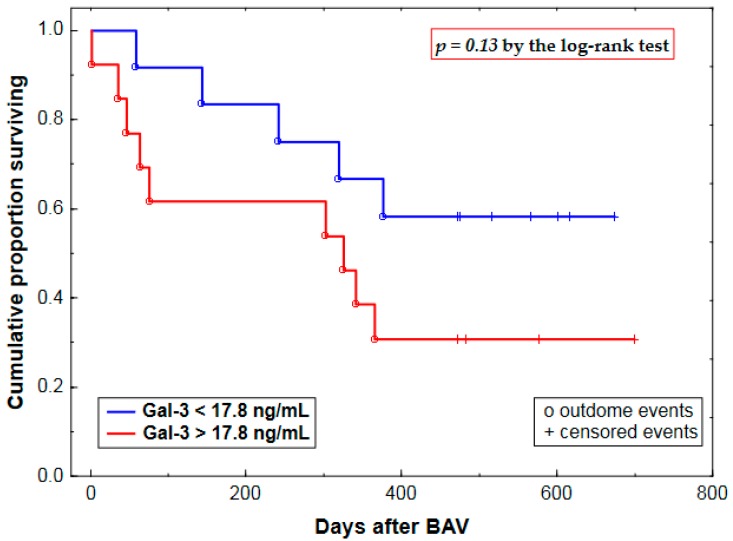
Kaplan–Meier survival curves by pre-procedural plasma galectin-3 (Gal-3) for aortic stenosis patients undergoing balloon aortic valvuloplasty (BAV).

On a univariate analysis limited to 25 AS patients undergoing BAV, pre-procedural Gal-3—categorized by a cut-off value of 17.8 ng/mL—tended to predict mortality (14 deaths) during a median follow-up of 362 days (Table 6, Figure 3). This relationship was strengthened after controlling for eGFR and Zva (Table 6), the only remaining predictors that entered the multivariate regression model (Zva: mean hazard ratio (HR) per rise of 0.5 mmHg/(mL/m^2^): 1.15 (95% confidence interval, 1.03–1.27), *p* = 0.01; eGFR: HR per decrease of 10 mL/min per 1.73 m^2^: 0.80 (0.59–1.07), *p* = 0.14).

The significance was maintained when other potential confounders were forced into regression models as covariates, including age, gender, NYHA functional class, AVA, EF, coexistent coronary artery disease or diabetes, and NT-proBNP levels. 

## 3. Discussion

We identified depressed eGFR as a strong determinant of circulating Gal-3 levels in patients with symptomatic degenerative AS referred for invasive treatment. In the study group as a whole, Gal-3 weakly predicted total mortality, nevertheless, the effect was abolished upon adjustment for eGFR. However, multivariate-adjusted pre-procedural Gal-3 was associated with worse survival regardless of the degree of renal dysfunction in AS subjects undergoing BAV.

To the best of our knowledge, our preliminary report is the first to demonstrate a prognostic value of Gal-3 in patients after BAV. With regard to post-TAVI subjects, there are conflicting results in terms of prognostic effects of pre-procedural Gal-3. According to Baldenhofer et al. [21], in 101 patients after TAVI, pre-procedural Gal-3 predicted both all-cause mortality and cardiovascular events over one year, which was not affected upon multivariate adjustment, including eGFR and NT-proBNP. On the other hand, Liebetrau et al. [23] described a prognostic effect of post-procedural but not pre-procedural Gal-3 on one-year mortality in 184 subjects undergoing TAVI, which suggests a role of unidentified peri-procedural factors. In addition, although Lindman et al. [22] observed a univariate association of pre-procedural Gal-3 with mortality over a mean follow-up of about 2 years in 345 subjects with severe AS referred for valve replacement (via transcatheter or surgical approach), nonetheless, the significance of the effect was lost on multivariate analysis.

### 3.1. Relations between Plasma Gal-3 and Renal Function—Mechanisms and Prognostic Implications

Our results supplement existing evidence of the close association of Gal-3 with eGFR in patients with HF [10,11,19], subjects recruited from the general population [13,16], and—recently—also AS subjects [20,21,22]. Baldenhofer et al. [21] reported a highly significant negative correlation of Gal-3 with eGFR in subjects with severe AS referred for TAVI., Additionally, in 583 patients with at least mild AS (out of whom 53% presented severe AS), Arangalage et al. [20] demonstrated that depressed creatinine clearance and increased age were the strongest determinants of plasma Gal-3. 

A predominant role of eGFR for circulating Gal-3 is well proven. Tang et al. [10] confirmed a close connection between high plasma Gal-3 and decreased eGFR in HF, while no relations between Gal-3 and indices of LV structure or function, either systolic or diastolic, were observed. Furthermore, Gopal et al. [19] have pointed out that eGFR was the only independent predictor of Gal-3 irrespective of the presence of HF, LV systolic function and compensation state. Notably, the strong curvilinear inverse relationships between Gal-3 and eGFR were superimposable for patients with and without HF [19], which suggests that renal impairment, but not HF by itself, was the predominant determinant of Gal-3 in that setting. Therefore, it can be hypothesized that the eGFR–Gal-3 relationship obscured expected associations of Gal-3 with other parameters also in our AS patients, in whom Gal-3 was unrelated to indices of stenosis severity, EF, LV mass and symptomatic status, like in the previously cited reports [20,21].

Mechanisms of excessive accumulation of Gal-3 in renal dysfunction have not been fully elucidated so far. In HF, Meijers et al. [26] demonstrated unchanged urinary Gal-3 excretion despite elevated plasma Gal-3 concentrations, and a 40% lower renal Gal-3 clearance, averaging only about 4 mL/min in the healthy controls, which is indicative of abnormal renal handling of Gal-3 in HF beyond eGFR depression.

In our study group as a whole, adjustment for eGFR abolished the association of baseline Gal-3 with mortality. This is in accordance with previous reports which have even suggested that the prognostic value of Gal-3in HF may be due to its association with renal dysfunction [27,28]. Additionally, in LURIC study participants hospitalized for coronary angiography, Gal-3 predicted all-cause mortality only in subjects with impaired renal function [29]. Of note, adjustment for baseline eGFR moderately attenuated the prognostic ability of Gal-3 with regard to the risk of new-onset HF in the Framingham Offspring Cohort, while the respective association lost its significance after controlling for development of incident chronic kidney disease (CKD) [13]. Moreover, an additional analysis of subjects without prevalent CKD revealed an independent contribution of baseline Gal-3 to a rapid renal function decline or incident CKD, irrespective of baseline eGFR [30]. Thus, the prognostic ability of Gal-3 appears partially mediated not only by its association with eGFR but also with progressive deterioration of renal function.

As far as the prognostic effect of eGFR in AS is concerned, baseline renal failure potently predicted 6–18-month mortality in 262 patients undergoing BAV [31], 274 medically treated inoperable subjects (out of whom 65% underwent BAV) [32], as well as in large groups of patients after TAVI [33,34] or SVR [35]. Of note, post-discharge worsening of renal function, measured at 30 days after discharge, independently predicted one-year mortality in 168 post-TAVI patients [36]. Thus, that the prognostic effect of Gal-3 in our patients undergoing BAV was observed after multivariate adjustment, including eGFR, does not allow to entirely exclude renal-dependent mechanisms as potential contributors to increased mortality in patients with higher pre-procedural Gal-3.

Admittedly, renal effects of Gal-3 are far from being unequivocally clarified. In various experimental models, upregulation of Gal-3 in response to kidney injury was linked to either a pro-resolution role or a pro-fibrotic effect, which translated into protection from injury [37,38,39] or exacerbated renal damage [40,41,42], respectively, the latter also including an animal AS model [43]. However, even assuming progressive renal function deterioration as a possible mechanism responsible for the detrimental prognostic effect of baseline Gal-3, this association would be expected to occur irrespective of treatment strategy, whereas Gal-3 independently predicted mortality exclusively in post-BAV patients. These results suggest that circulating Gal-3 might reflect BAV-related pathophysiological mechanisms that are linked to adverse outcome preferentially after BAV.

### 3.2. Mechanistic Considerations—Valvular, Myocardial and Vascular Effects of Gal-3 as Potential Contributors to the Prognostic Ability of Pre-procedural Plasma Gal-3 in AS Patients Undergoing BAV

A high restenosis rate has a profound impact on prognosis in post-BAV patients. In a histological study of aortic valves excised from patients operated upon 1–36 months after BAV, mainly due to clinical restenosis, van den Brand et al. [44] demonstrated—in 11 out of 12 study subjects—the presence of a non-collagenous young scar tissue filling up tears and lacerations in the dense collagenous valve stroma, fractured calcified nodules and splits between commissures up to 24 months after valve dilatation, none of which were observed in control stenotic aortic valves with no preceding BAV. Those findings are indicative of a prolonged scarring reaction with consequent high interindividual variability of the time-curse of scar organization and collagenisation, i.e., processes involved in restenosis. Accordingly, because the cell-rich young scar tissue is composed of fibroblasts, inflammatory cells and sprouting capillaries [44], it can be hypothesized that subjects with higher pre-procedural Gal-3—whose binding sites were found predominantly in fibroblasts and extracellular matrix [1]—can be more prone to accelerated fibroblasts proliferation, collagen synthesis and subsequent restenosis.

Admittedly, circulating pre-procedural Gal-3 may not necessarily reflect local Gal-3 levels within the aortic valve. However, Sadaba et al. [45] reported a moderate positive correlation between serum Gal-3 and Gal-3 expression in valvular interstitial in patients with severe AS undergoing SVR. Moreover, valvular Gal-3 colocalized also with inflammatory and osteogenic markers in those patients, and in vitro Gal-3 induced pro-inflammatory, pro-fibrotic and osteogenic effects in valvular interstitial cells [45]. These associations are probably even more operative after BAV, known to be associated with an inflammatory response, a well-recognized stimulus for further Gal-3 upregulation. Therefore, an independent association of pre-procedural plasma Gal-3 with mortality in post-BAV patients could reflect a role of local intravalvular Gal-3 generation in the development of restenosis.

On the other hand, our results might also result from a contribution of myocardial Gal-3 to collagen deposition and consequent myocardial dysfunction. LV fibrosis, quantified my magnetic resonance imaging, predicted mortality during a median follow-up of 2.9 years in 194 AS patients undergoing SVR or TAVI [46]. Indeed, pharmacological inhibition of Gal-3 activity prevented tumor growth factor-β (TGF-β) upregulation, LV hypertrophy, fibrosis, and diastolic dysfunction in aldosterone/salt-treated rats, thereby mimicking effects of spironolactone [3]. Furthermore, genetic disruption of Gal-3 formation or Gal-3 inactivation protected against LV fibrosis and dysfunction and improved survival in various experimental models of LV pressure overload [2,4]. Moreover, Gal-3 overexpression preceded the onset of HF in failure-prone rats [1], which supports the notion that Gal-3 might accelerate the transition from compensated hypertrophy into overt HF by the stimulation of perivascular and interstitial myocardial fibrosis via the TGF-β/Smad3 signaling pathway [47,48].

Nevertheless, conclusions from experimental models cannot be easily extrapolated into clinical conditions, and clinical correlates of blood Gal-3 differ between various clinical settings. Although plasma Gal-3 at Day 7 was related to myocardial extracellular volume fraction at six months in patients after acute myocardial infarction [49], other reports on the association of baseline Gal-3 with LV remodeling brought contradictory results [50,51,52]. As to severe AS, circulating Gal-3 did not correlate with percent collagen area or the number of macrophages in tissue samples collected from the basal interventricular septum at the time of valve surgery for AS [22]. In accordance with this finding, plasma Gal-3 did not predict the onset of symptoms over a mean follow-up of three years in 330 asymptomatic AS patients [20]. Such an association would be expected, if Gal-3 significantly contributed to LV dysfunction in AS. Therefore, in line with the previously cited report of Sadaba et al. [45], circulating Gal-3 might reflect intravalvular rather than myocardial Gal-3 content in AS.

Beyond valvular, myocardial and renal effects, Gal-3 upregulation in vascular myocytes may be accountable for the aldosterone-induced vascular fibrosis [53], which can explain a positive correlation—irrespective of blood pressure and eGFR—between Gal-3 and pulse wave velocity in the general population [54]. Because higher arterial stiffness contributes to LV afterload via augmented central systolic blood pressure, this might be an alternative pathway facilitating HF development in subjects with high Gal-3 levels. In fact, depressed systemic arterial compliance accompanying moderate-to-severe AS was previously linked to LV systolic and diastolic dysfunction [55], and to the severity of HF symptoms [56]. Moreover, associations of baseline Zva, a measure of global LV afterload, with two-year mortality were demonstrated in AS patients undergoing TAVI [57], SVR [58] or treated medically [58]. In our hands, Zva predicted mortality in AS subjects undergoing BAV. However, because the prognostic ability t of Gal-3 in these post-BAV patients was observed after multivariate adjustment including Zva, the effect of Gal-3 appears independent of Zva. 

Gal-3 might also accelerate atherogenesis, thus contributing to adverse outcome. However, data on a potential role of Gal-3 in the development of plaques are inconsistent, and opposite effects were described in various models of experimental atherosclerosis [59,60]. As a multifunctional protein, Gal-3 can interfere with a variety of pathways and large-scale studies have not confirmed independent associations of Gal-3 with the risk of cardiovascular ischemic events [61,62].

### 3.3. Mechanistic Considerations—Summary

An independent association of Gal-3 with mortality only in post-BAV patients suggests an underlying mechanism that is limited to AS subjects undergoing BAV, i.e., contribution of Gal-3 to development of valve restenosis and subsequent HF recurrence. In contrast, alternative mechanisms would be expected to take place in all of the study subjects irrespective of type of treatment employed, including Gal-3 involvement in renal function decline, myocardial fibrosis and increased vascular stiffness with consequent progressive HF. However, the absence of an independent prognostic effect of Gal-3 for the whole study group argues against these mechanisms. Nevertheless, further investigations are warranted to validate the proposed concept in a larger group of AS patients.

## 4. Materials and Methods

### 4.1. Patients

We studied 80 consecutive patients (mean age, 79 ± 8 years; 44 women and 36 men) with the final diagnosis of severe or moderate symptomatic degenerative AS (defined as a calculated AVA index ≤0.9 cm^2^/m^2^ body-surface area or mean transvalvular aortic pressure gradient ≥25 mmHg [24]) referred for invasive treatment to our tertiary care center between December 2013 and August 2014.

The study was conducted in accordance with the Declaration of Helsinki, and the protocol was approved by the Bioethics Committee of Jagiellonian University (Approval number: KBET/257/B/2013 dated 23 October 2013). All subjects gave their informed consent for inclusion to the study.

### 4.2. Study Protocol

Demographic and clinical patients’ characteristics, including cardiovascular risk factors and HF symptoms graded by New York Heart Association (NYHA) functional classification were registered at admission. Maximal and mean transvalvular aortic pressure gradients were derived by the modified Bernoulli formula from continuous Doppler recordings and AVA index was calculated by the standard continuity equation. LV volumes and EF were computed by the biplane method of disks summation (modified Simpson’s rule), as currently recommended [63], and LV mass index was assessed by means of the modified Devereux formula based on M-mode measurements at end-diastole [63,64].

In addition to routine echocardiographic parameters of AS and LV structure and function, Zva, an index of global LV afterload, was computed from brachial systolic pressure, mean transvalvular pressure gradient and LV volumes as proposed previously [55,56,58].

Blood was drawn from an antecubital vein for routine biochemical analyses and extended assays. Gal-3 was measured by means of an automated enzyme-linked fluorescent assay (VIDAS Galectin-3 assay, bioMerieux, Marcy l’Etoile, France) (the lower detection limit, 2.4 ng/mL; coefficient of variation <6%) [65] and NT-proBNP by an electrochemiluminescent immunoassay (Elecsys proBNP, Roche Diagnostics GmbH, Mannheim, Germany) (the lower detection limit, 5 pg/mL; coefficient of variation <4%). eGFR was calculated according to the Chronic Kidney Disease Epidemiology Collaboration formula [25].

The study subjects underwent a complex diagnostic workup in our center. The therapeutic strategy was chosen following a dedicated multidisciplinary team (Heart Team)-oriented approach, including SVR, TAVI (patients unsuitable for conventional surgery or with high surgical risk) and BAV (a palliative measure, when both SVR and TAVI contraindicated, or as a bridge to TAVI or SVR) [24]. Those considered ineligible for invasive procedures received optimal medical therapy. After discharge, our patients or their relatives were contacted by telephone in order to ascertain vital status of the study subjects.

### 4.3. Statistical Analysis

Continuous variables were presented as mean and standard deviation (SD) or median and interquartile range for non-normally distributed data. The accordance with a Gaussian distribution was checked by the Liliefors test. Categorical data were shown as numbers and percentages. Bivariate correlations between patients’ characteristics and levels of Gal-3 and NT-proBNP were estimated by Pearson’s or Spearman’s correlation coefficients (*r*) for continuous and ordinal variables, respectively. Gal-3 and NT-proBNP concentrations were natural logarithmically (log) transformed owing to their right-skewed distributions. Intergroup comparisons were performed by 2-tailed Student’s *t*-test and chi-square test for continuous and categorical variables, respectively.

The predictive ability of Gal-3 was tested by comparing Kaplan–Meier survival curves by the log-rank test with Gal-3 levels categorized according to a cut-off value of 17.8 ng/mL, previously proposed for risk stratification in patients with established HF [5,11]. Deaths from any cause were considered as outcome events. Additionally, Cox proportional hazards regression was applied to assess determinants of the risk of all-cause mortality in the study subjects. For continuous covariates, mean HR corresponds to a relative mortality risk associated with each change in the predictor variable by a given value or 1 SD. As to dichotomous explanatory variables (i.e., factors, e.g., Gal-3 over 17.8 ng/mL), HR reflects a proportional mortality risk in the subjects exposed to a factor of interest in comparison to those without such exposure. Cox modeling was repeated after adjustment for eGFR in order to exclude the eGFR–Gal-3 relationship as a cause of the prognostic value of Gal-3 in a univariate analysis. The proportionality assumption of the Cox method was validated by confirming the lack of significant prognostic effects for interaction terms between each covariate and time. A *p*-value below 0.05 was assumed significant; Bonferroni correction for multiple testing was used with regard to correlates of Gal-3 and NT-proBNP levels and intergroup comparisons according to treatment modality.

## 5. Conclusions

In degenerative AS, plasma Gal-3 is mainly determined by the degree of coexistent renal insufficiency, being independent of symptomatic status, stenosis severity, valvulo-arterial impedance and LV hypertrophy or dysfunction. The inverse eGFR–Gal-3 relationship underlies a weak association between Gal-3 and adverse outcome in patients with degenerative AS referred for invasive therapy irrespective of type of treatment employed. In contrast, multivariate-adjusted pre-procedural Gal-3 appears an independent mortality predictor in high-risk AS patients undergoing BAV.

## Figures and Tables

**Table 1:** Demographic and clinical characteristics of the study patients.

Age, years	79 ± 8
Gender, male	36 (45%)
Body mass index, kg/m^2^	26.5 ± 4.1
NYHA functional class, II/III/IV	40/25/15 (50/31/19%)
eGFR, mL/min per 1.73 m^2^	58 ± 21
Hypertension	71 (89%)
Diabetes mellitus	26 (33%)
Coronary artery disease	36 (45%)
NT-proBNP, pg/mL	2554 (1168–5244)

Data are shown as mean ± SD, median (interquartile range) or n (%). Abbreviations: eGFR, estimated glomerular filtration rate by the Chronic Kidney Disease Epidemiology Collaboration equation [25]; NYHA, New York Heart Association; NT-proBNP, N-terminal pro-B-type natriuretic peptide.

**Table 2:** Echocardiographic parameters, systolic blood pressure and valvulo-arterial impedance.

AVA, cm^2^	0.7 ± 0.2
AVA index, cm^2^/m^2^	0.4 ± 0.1
Maximal transvalvular aortic pressure gradient, mmHg	84 ± 30
Mean transvalvular aortic pressure gradient, mmHg	52 ± 21
LVMI, g/m^2^	174 ± 55
EF, %	52 ± 15
Stroke volume index, mL/m^2^	34.0 ± 11.6
Systolic blood pressure, mmHg	129 ± 20
Zva, mmHg/(mL/m^2^)	5.9 ± 2.3

Data are shown as mean ± SD. Abbreviations: AVA: aortic valve area; EF: left ventricular ejection fraction; LVMI: left ventricular mass index; Zva, valvulo-arterial impedance.

**Table 3:** Relations between patients’ characteristics and log-transformed plasma levels of galectin-3 (Gal-3) and N-terminal pro-B-type natriuretic peptide (NT-proBNP).

Characteristic	Age	eGFR	NYHA	AVA	MPG	LVMI	EF	Zva
**Gal-3**	0.09 0.4	−0.61 * <0.001	0.16 0.2	0.01 0.9	−0.25 0.02	0.17 0.14	−0.12 0.3	−0.07 0.5
**NT-proBNP**	0.09 0.4	−0.37 * 0.001	0.20 0.07	−0.17 0.14	0.18 0.11	0.40 * <0.001	−0.26 0.02	−0.06 0.6

Data are shown as Pearson’s or Spearman’s correlation coefficients (*r*) for continuous and ordinal characteristics, respectively, and associated *p* values. * Significant after Bonferroni correction for multiple comparisons. Abbreviations: AVA: aortic valve area; EF: left ventricular ejection fraction; eGFR: estimated glomerular filtration rate; LVMI: left ventricular mass index; NYHA: New York Heart Association functional class; MPG: mean transvalvular aortic pressure gradient; Zva: valvulo-arterial impedance.

**Table 4:** Patients’ characteristics according to treatment modality.

Characteristic	Balloon Aortic Valvuloplasty (*n* = 25)	Other Treatment Modalities (*n* = 55)	*p*
Age, years	84 ± 6	78 ± 8	<0.001 *
Gender, male	11 (44%)	25 (45%)	0.9
Body-mass index, kg/m^2^	25.3 ± 3.7	27.0 ± 4.2	0.1
NYHA functional class, II/III/IV	8/8/9 (32/32/36%)	32/17/6 (58/31/11%)	0.02
Hypertension	24 (96%)	47 (85%)	0.2
Diabetes mellitus	12 (48%)	14 (25%)	0.05
Coronary artery disease	14 (56%)	22 (40%)	0.2
NT-proBNP, pg/mL	3635 (2349–5755)	2294 (1004–5055)	0.14
AVA index, cm^2^/m^2^	0.3 ± 0.1	0.4 ± 0.1	0.05
Maximal transvalvular aortic pressure gradient, mmHg	83 ± 28	84 ± 32	0.9
Mean transvalvular aortic pressure gradient, mmHg	52 ± 19	52 ± 22	0.9
LVMI, g/m^2^	183 ± 37	170 ± 61	0.3
EF, %	48 ± 16	53 ± 15	0.2
Stroke volume index, mL/m^2^	32.2 ± 11.9	34.8 ± 11.4	0.4
Systolic blood pressure, mmHg	128 ± 22	130 ± 19	0.7
Zva, mmHg/(mL/m^2^)	6.4 ± 2.7	5.7 ± 2.0	0.2
Galectin-3 (ng/mL)	19.0 (13.1–25.2)	14.5 (11.5–19.5)	0.06

Data are shown as mean ± SD, median (interquartile range) or *n* (%). * Significant after Bonferroni correction for multiple comparisons. Abbreviations are the same as in Table 1 and Table 2.

**Table 5:** Cox proportional hazards regression of all-cause mortality on baseline galectin-3 (Gal-3) levels for all AS patients irrespective of type of treatment employed.

Predictor Variable	β ± SEM	Hazard Ratio (HR)	
		Mean HR (95% CI)	*p*
Gal-3, unadjusted			
Gal-3 (Gal-3 >17.8 vs. <17.8 ng/mL)	0.71 ± 0.43	2.03 (0.88–4.69)	0.09
Gal-3 (per 1-SD increment)	0.40 ± 0.20	1.49 (1.00–2.21)	0.05
Gal-3, adjusted for eGFR			
Gal-3 (Gal-3 >17.8 vs. <17.8 ng/mL)	0.53 ± 0.52	1.70 (0.61–4.73)	0.31
Gal-3 (per 1-SD increment)	0.38 ± 0.29	1.46 (0.83–2.57)	0.19

Abbreviations: AS: aortic stenosis; β: regression coefficient; CI: confidence interval; eGFR: estimated glomerular filtration rate; SD: standard deviation; SEM: standard error of the mean.

**Table 6:** Cox proportional hazards regression of all-cause mortality on pre-procedural galectin-3 (Gal-3) levels for AS patients undergoing balloon aortic valvuloplasty.

Predictor Variable	β ± SEM	Hazard Ratio (HR)	
		Mean HR (95% CI)	*p*
Gal-3, unadjusted			
Gal-3 (Gal-3 >17.8 vs. <17.8 ng/mL)	0.84 ± 0.56	2.32 (0.78–6.96)	0.13
Gal-3, adjusted for eGFR			
Gal-3 (Gal-3 >17.8 vs. <17.8 ng/mL)	1.19 ± 0.69	3.30 (0.86–12.7)	0.08
Gal-3, adjusted for eGFR and Zva			
Gal-3 (Gal-3 >17.8 vs. <17.8 ng/mL)	2.00 ± 0.81	7.41 (1.52–36.1)	0.01

Zva: valvulo-arterial impedance; other abbreviations are the same as in Table 5.

## Abbreviations

AS	Aortic stenosis
AVA	Aortic valve area
BAV	Balloon aortic valvuloplasty
CKD	Chronic kidney disease
CI	Confidence interval
EF	Left ventricular ejection fraction
eGFR	Estimated glomerular filtration rate
Gal-3	Galectin-3
HF	Heart failure
HR	Hazard ratio
LV	Left ventricular
NT-proBNP	N-terminal pro-B-type natriuretic peptide
NYHA	New York Heart Association
MPG	Mean transvalvular aortic pressure gradient
SD	Standard deviation
SEM	Standard error of the mean
SVR	Surgical aortic valve replacement
TAVI	Transcatheter aortic valve implantation
TGF-β	Tumor growth factor-β
Zva	Valvulo-arterial impedance
